# Preliminary report on a study of health-related quality of life in patients with rheumatoid arthritis

**DOI:** 10.1007/s00296-012-2421-5

**Published:** 2012-03-28

**Authors:** Krzysztof Kanecki, Piotr Tyszko, Małgorzata Wisłowska, Justyna Łyczkowska-Piotrowska

**Affiliations:** 1 Rheumatology and Internal Medicine Department, Central Clinical Hospital, Ministry of Internal Affairs and Administration, ul. Wołoska 137, 02-507 Warsaw, Poland; 2 Department of Health Care, Medical University of Warsaw, ul. Oczki. 3, 02-007 Warsaw, Poland

**Keywords:** Rheumatoid arthritis, Health-related quality of life, SF-36

## Abstract

There are studies about health-related quality of life (HRQoL) in patients with rheumatoid arthritis (RA), but few studies prospectively assessed HRQoL. The main purpose of this study was to analyze HRQoL in patients hospitalized due to RA exacerbation and observed over a planned 2-year follow-up in an outpatient setting. The study involved 42 women and 9 men, at mean age of 62.5 years (SD ± 12.6). The mean duration of the study was 22–23 months. The HRQoL analysis was performed using the SF-36 survey. At the beginning of the study, basic data on age, sex, selected biochemical (ESR, CRP, GFR, hemoglobin, plasma albumin, plasma protein), and clinical parameters (the duration of RA, VAS, DAS28, BMI, the presence of cardiovascular disease, diabetes, osteoporosis, osteoporotic fractures, osteoarthritis, neoplasm) were collected. Questionnaires were completed at the beginning and end of the study. Statistically significant reductions in HRQoL scores were observed in social functioning (SF; 0.42 vs 0.32, *P* < 0.05), whereas role-emotional health (RE; 0.48 vs 0.59, *P* < 0.05) and mental health (MH; 0.47 vs 0.54, *P* < 0.05) scores were increased. A decrease in the SF was positively correlated with the lack of osteoporosis at baseline (*r* = 0.35, *P* > 0.02). An increase in the MH was inversely correlated with BMI (*r* = −0.31, *P* < 0.05), and the level of hemoglobin (*r* = −0.32, *P* < 0.028) and positively correlated with the presence of osteoarthritis at baseline (*r* = 0.29, *P* < 0.05). In RA patients, dimensions of HRQoL as SF, RE, and MH could change within 2 years and these changes could be related to comorbidities. Although preliminary findings are promising, further studies are needed.

## Introduction and purpose

Rheumatoid arthritis (RA) is a systemic, autoimmune, inflammatory disease of unknown etiology [1]. RA is usually characterized as a progressive disease, leading to joint destruction, physical activity limitation, disability, and premature death. Other complications of the disease are a worsening of social functioning, emotional health, and the quality of life. Additional relevant factors connected with treatment are the costs of health care of RA patients and the costs of complications due to RA treatment. The need for a comprehensive approach to the evaluation of disease progression resulted in an increased interest in an objective assessment of disease progression and patient’s subjective well-being. The attention to the health-related quality of life is a consequence of a comprehensive approach to treating patients with RA. The patients’ quality of life may be affected by many factors in multiple dimensions. In the case of patients with RA, additional factors that may modify HRQoL are disability, pain, fatigue, depression, comorbidities and others.

HRQoL estimation with use of questionnaires can play important role in treatment of RA. In literature, it was reported that a RAPID3 or a RADAI5 score could serve the same function as ESR and CRP for rheumatologists and a RADAI5 requires minimal cost and professional time [2]. Patient questionnaire scores for physical function are the most significant predictors of work disability and premature mortality, somewhat greater than joint count measures, and far more significant than radiographic and any laboratory data [3]. Additionally, it was reported that patient questionnaire scores indicating poor functional status predicted survival of less than 50 % of RA patients over the next 5 years [4]. The questionnaires most commonly used in assessing HRQoL in RA patients are the following: AIMS (Arthritis Impact Measurement Scales) [5], RAQoL (Rheumatoid Arthritis Quality of Life instrument) [6], HAQ (Health Assessment Questionnaire) [7], and SF-36 (36-item short-form health survey) [8].

There are studies analyzing HRQoL with SF-36 survey among patients with RA. SF-36 was described as reliable, valid, and able to measure the clinically important aspects of health status in British patients with RA [9]. The Chinese version of the SF-36 showed reasonable reliability, criterion validity, and responsiveness with limitations in certain subscales in RA patients [10]. Small improvements in HRQoL measured with SF-36 survey were seen in cross-sectional and longitudinal studies in RA patients from Sweden, and these improvements were exclusively attributable to treatment with biologics [11]. In one systematic review of the literature and meta-analysis assessing the effect of biotherapies versus placebo on fatigue, in combination with DMARDs with use of (FACIT-F) or short-form 36 (SF-36) vitality scores at baseline and at week 24, it was reported that the impact of biotherapies on fatigue in RA was small [12]. In study focused on HRQoL in patients with severe osteoarthritis or rheumatoid arthritis, an arthritis-specific health index (ASHI) was used [13]. The patterns of correlations between SF-36 scales and the ASHI were very similar across developmental and cross-validation samples. In conclusion, it was said that SF-36 survey can be useful in studies of rheumatoid arthritis. Data from 7 double-blind, randomized controlled trials that examined the effectiveness of one or more interventions in RA and the primary outcome measures evaluating data from SF-36 surveys showed that SF-36 deserves serious consideration for inclusion in the core set of outcomes in RA trials [14].

The primary aim of this study was to analyze changes in SF-36 scale scores over a 2-year period. A secondary aim was to analyze the correlation between the observed changes in HRQoL scale scores and the demographic, biochemical, and clinical parameters. Another additional aim was psychometric evaluation of the HRQoL instrument used in this study in terms of its accuracy and reliability. To assess HRQoL, we used SF-36 questionnaires completed by the patients. Questionnaires should be evaluated as to their psychometric reliability, accuracy, and the ability to capture changes in the evaluated parameters in a given period of time [15]. This evaluation was done in this study. Standardization of the obtained results, that is, presenting them within the framework of the adopted scale, usually requires their conversion into a 0–1 or 0–100 % format and allows a comparison of the results not only in the study group but also between different groups of patients. In this paper, the obtained results were presented in the 0–1 range. The psychometric assessment of SF-36 was calculated using Cronbach’s alpha reliability coefficients and parameters of convergent validity and discriminant validity.

## Materials and methods

HRQoL was studied in patients of the Rheumatology and Internal Medicine Department of the Central Clinical Hospital of Ministry of Internal Affairs and Administration in Warsaw. In the study, HRQoL analysis was based on data obtained from 51 patients: 42 women and 9 men. In the study group, 35 patients (pts) had cardiovascular disease, 6 pts had diabetes, 20 pts had osteoporosis, 44 pts had osteoarthritis, 8 were treated because of neoplastic disease within the last 5 years, and 10 pts had previous osteoporotic fractures. RA patients were treated with a standard treatment regimen. Median RA duration was 10 years, 42 pts had erosive disease, 35 pts were treated with MTX (25 monotherapy, 10 in combination with other DMARDs), 9 pts were treated with anti-TNF, 2 pts with tocilizumab, 1 pts with rituksymab; 41 pts took low dose of oral steroids. Licenses for the use of SF-36 were achieved (license numbers CT113991/OP000073 and CT134122/OP013928). This study was a continuation of previous work focused of HRQoL in RA patients [16]. Socio-demographic characteristics, medical records, and HRQoL data collected at baseline were presented in the previous study. The next completion of the HRQoL questionnaire was to take place after 2 years. In this study, the second completion of the HRQoL questionnaire was conducted after a mean period of 22–23 months, in person (preferred option) or via telephone. The baseline data collection was done between April 2009 and December 2009, and the second HRQoL assessment was done between April 2011 and June 2011. The HRQoL scales were measured according to recommendations by the authors of the SF-36 survey. The data on HRQoL were collected using version 2 of the SF-36 survey, with the text translated into Polish provided by the Licensor of the SF-36 survey. The collected data were analyzed using Statistica program, ver. 6. Statistical significance of changes in SF-36 scale scores was tested using nonparametric statistics (Wilcoxon’s tests) for paired factors. The level of statistical significance was set at *P* < 0.05. The HRQoL measurement tool—the SF-36 survey includes 36 items (questions) forming 8 scales. Abbreviations of scales used in tables: PF—Physical Functioning, RP—Role-Physical, BP—Bodily Pain, GH—General Health, VT—Vitality; SF—Social Functioning, RE—Role-Emotional, MH—Mental Health, NS—no statistical significance.

## Results

This study analyzed changes in SF-36 scale scores. The study group consisted of 51 patients, 42 women and 9 men. The average age of all patients at baseline was 62.5 years (SD ±12.6 years), maximum 86 years, minimum 29 years, median 63 years. After 22–23 months, HRQoL data were collected from 47 patients; 3 people died during the study and one patient’s clinical condition did not allow for HRQoL data collection. Table 1 shows the results of the SF-36 scales measurement in a standardized form. The first measurement (meas. I) refers to the HRQoL scales assessed at baseline, the second measurement (meas. II) refers to the HRQoL scales after the follow-up period. Standardization of the HRQoL scales involved presenting the individual scores in the form of values falling within an accepted range, either in the percentage form, or in the range from 0 to 1, with low values indicating poor HRQoL in the given dimension, or worse well-being of the patient. Conversely, high values indicate improved HRQoL, or better condition of the patient. The values of all HRQoL scale scores were below the half of their corresponding maximum values. In general, patients well-tolerated the study and provided prompt responses during their SF-36 survey completion. The psychometric value of the SF-36 questionnaire was evaluated with respect to its reliability and validity. The reliability of the questionnaire was assessed using an internal consistency test (Cronbach’s alpha) [17]. Calculated in the program Statistica, Cronbach’s alpha values for individual scales were higher than 0.7, with the PF scale value above 0.9. It has been pointed out in literature that an instrument meeting these criteria is of good reliability [18]. In this study, Cronbach’s alpha values indicate a good reliability of the SF-36 form. The validity of the questionnaire was analyzed using the parameters of convergent validity and discriminant validity. The psychometric assessment parameters of data collected during the second measurement (presented in Table 2) show good results for the individual scales, despite the fact that values of 100 % were not achieved in all scales. The results show good validity of the SF-36 questionnaire.

**Table 1:** Statistical characteristics of SF-36 scales

SF-36 scales	Mean value			Minimum value		Maximum value		Standard deviation		Median	
	Meas. I	Meas. II	Statistical significance	Meas. I	Meas. II	I Meas.	II Meas.	I Meas.	II Meas.	I Meas.	II Meas.
PF	0.43	0.41	NS	0.0	0.0	1.0	0.85	0.26	0.19	0.40	0.40
RP	0.42	0.39	NS	0.0	0.0	0.87	0.75	0.16	0.17	0.44	0.38
BP	0.32	0.39	NS	0.0	0.0	1.0	0.84	0.18	0.20	0.32	0.41
GH	0.29	0.30	NS	0.0	0.0	0.62	0.77	0.15	0.17	0.30	0.25
VT	0.32	0.32	NS	0.15	0.0	0.8	0.63	0.12	0.13	0.30	0.31
SF	0.42	0.32	*P* < 0.05	0.0	0.0	1.0	0.75	0.26	0.28	0.50	0.25
RE	0.48	0.59	*P* < 0.05	0.0	0.0	1.0	0.92	0.24	0.19	0.50	0.67
MH	0.47	0.54	*P* < 0.05	0.24	0.0	0.68	0.75	0.10	0.13	0.48	0.55

**Table 2:** The psychometric assessment of SF-36

SF-36 scales	Cronbach’s alpha	Convergent validity test (%)	Discriminant validity test (%)
PF	0.90	100	96
RP	0.85	100	89
BP	0.82	100	79
GH	0.79	100	89
VT	0.72	100	93
SF	0.91	100	100
RE	0.79	100	100
MH	0.76	80	80

In the analyzed period of time, a statistically significant reduction in HRQoL scale scores was observed in the SF scale (0.42 vs 0.32, *P* < 0.05). A statistically significant improvement in HRQoL scores was observed in the RE scale (0.48 vs 0.59, *P* < 0.05) and MH scale (0.47 vs 0.54, *P* < 0.05). There were no significant changes in other dimensions of HRQoL (PF, RP, BP, GH, VT). After 2 years, HRQoL scale scores showed statistically significant changes. Moreover, statistically significant correlations with selected biochemical and clinical variables were recorded. A decrease in SF scale score was correlated with the lack of osteoporosis at baseline (*r* = 0.35, *P* < 0.02). An improvement in MH scale score was inversely correlated with body mass index (BMI) values (*r* = −0.31, *P* < 0.05) and baseline hemoglobin levels (*r* = −0.32, *P* < 0.028) and positively correlated with the presence of osteoarthritis at baseline (*r* = 0.29, *P* < 0.05).

## Discussion

A growing interest in the health-related quality of life in chronic diseases is illustrated by the number of studies available in the medical literature. The number of these publications has grown significantly in recent years. HRQoL in medicine was adopted to describe the effect of diseases and its treatment on physical, psychological, and social aspects of patients’ well-being. HRQoL is one of the components of quality of life, which can be affected by many factors, not only medical health, but also the cultural, socio-economic, and other factors as well as other areas of human activity. When we estimate the influence of numerous factors on the quality of life, we examine the influence of variables on patients’ subjective feelings and try to describe HRQoL objectively, and even using numbers. The subjective nature of HRQoL makes it difficult to analyze HRQoL and obtained data only approximately characterize HRQOL. An analysis of HRQOL may be helpful in building a comprehensive therapeutic strategy targeted to achieve the best possible physical, mental, and social well-being of the patient. In patients with rheumatoid arthritis, an assessment of HRQoL is the most important element in building the therapeutic strategy. This results from the fact that RA is often progressive in nature, leading to disability, as well as affecting life expectancy.

Many questionnaires, used to assess HRQOL, have been presented as reliable and studied in patient populations in different countries or different clinical situations [19, 20]. Several studies have indicated that the HRQoL of RA patients was significantly lower than that in the general population [21, 22]. It was also observed in Polish patients [23]. There are questionnaires specific to particular diseases or medical procedures as well as general questionnaires. A general questionnaire, the SF-36, was selected for this study because of its brevity, comprehensiveness, a high standard of integrity, and good reliability and validity parameters [24, 25]. The rationale behind selecting a general questionnaire for the study was the fact that patients with RA often suffer from other diseases that could hinder the assessment of HRQoL. In this paper, the SF-36 questionnaire was well accepted by patients. Reliability coefficients (Cronbach’s alpha) for the different scales were higher than 0.7 and, in the case of PF, above 0.9. In this study, accuracy parameters in most cases have reached 100 % in tests of consistency. Consistency tests analyze what proportion of responses to each question correlates with the value of the own scale at the level above 0, 4. The discriminant validity test in most cases yielded values of 80–100 %. A discriminant validity test describes what proportion of SF-36 items correlates higher with its own scale than with other scales.

The studied group of patients remained under specialist care during the study. Detailed patient education, systematic specialist outpatient care, and a regular use of medications were all part of a comprehensive approach to treatment of RA patients. This holistic approach was taken in order to slow the progression of the disease, to achieve the maximum reduction of RA-related complications, and to improve the patients’ functioning in a local community. In this study, we observed significant changes in HRQoL scores relating mainly to social activities or emotional and mental health. These scales seem to be more difficult to describe, as opposed to scales related to physical health. In this study, we expected to see an age-dependent decrease in physical functioning, as a deterioration in physical function with age has been reported in literature [26]. In the present study, there were no significant changes in HRQoL scale scores relating to physical functioning. This can be explained by systematic specialist outpatient care, regular treatment, and patient education. On the other hand, it was reported that an improvement in the area of emotional well-being in patients with RA can be related to age [27]. Increase in values of scale describing mental health also observed in this prospective 2-year follow-up study. These observations may be helpful in the treatment of patients, with a special focus on the elderly with RA. It has been reported in literature that a clinical remission in rheumatoid arthritis may reduce symptoms of depression and anxiety and that it independently improves the quality of life [28]. The use of therapies aimed at achieving clinical remission of RA may result in an improvement in mental, emotional areas of HRQoL.

In one study on HRQoL with use of SF-36, it was indicated that the appearance of comorbidities may have an impact on physical health domains, but no significant effect on the emotional health [29]. The presence of comorbidities in patients with RA in the beginning of the study may explain the lack of significant score changes in the SF-36 scales related to physical functioning.

There have been reports of a relationship between anemia and the well-being of RA patients [30]. In our study, we showed a statistically significant negative correlation between hemoglobin levels and changes in mental health (MH) scores. Baseline hemoglobin levels can be related to RA progression. It can be assumed that patients with lower baseline hemoglobin level may have advanced disease progression, physical activity limitations, and poor well-being. Improvement in mental health scores in these patients may be related to specialist outpatient care during the study. This conclusion is important according to report from other study that RA patients in comparison with the general population revealed a reduction of −0.27 for mental health measured by standardized difference scores (s-scores), indicating a low to moderate disease effect for mental health [31]. Mental health is very important factor in RA patients because depression is related to poor treatment compliance and increased morbidity and mortality in rheumatic conditions [32]. The presence of a significant negative correlation between the body mass index (BMI) and changes in mental health scale (MH) scores can be explained in a similar fashion. These results may require further research. In this study, the presence of osteoarthritis (OA) at baseline was significantly positively correlated with changes in MH scale scores. The additional presence of osteoarthritis indicates worse functioning in RA patients. One trial, comparing the well-being of RA patients with that of OA patients, showed no significant difference between the functioning and quality of life in the study groups, although the results suggested a better quality of life in the group of RA patients [33]. It is possible that the presence of OA and RA may widely worsen HRQoL at baseline. The comprehensive treatment and care implemented over 2 years can cause improvement in all HRQoL scales, but noticeable improvement in HRQoL mental health scale. The lack of osteoporosis at study baseline was significantly positively correlated with a decrease in the SF scale score. The lack of osteoporosis at baseline might be related to low RA activity, but the progression of the disease and the influence of drugs could increase the risk of osteoporosis and a deterioration of HRQoL in the SF scale.

## Conclusions

1. The prospective 2-year study on health-related quality of life (HRQoL) in RA patients under specialist outpatient care showed a statistically significant decrease in the value of the scale related to social functioning (SF), increase in the value of the scales related to the role-emotional problems (RE) and increase in the value of the scale related to mental health (MH).
2. The prospective 2-year study on health-related quality of life in patients with RA under specialist outpatient care showed no statistically significant score changes in the HRQoL scales related to physical functioning (PF), role-physical (RP), bodily pain (BP), general health perception (GH), and vitality (VT).
3. The SF-36 survey was shown to have good psychometric assessment parameters, indicating its usefulness in the research on HRQoL in RA patients. In addition, the SF-36 questionnaire was well accepted by patients.
4. In the present study, some HRQoL scales were significantly correlated with selected biochemical and clinical variables. Deterioration in the social function scale (SF) score was positively correlated with the lack of osteoporosis at baseline. An improvement in the mental health scale (MH) score was inversely correlated with the values of body mass index and hemoglobin levels at baseline and positively correlated with the presence of osteoarthritis at baseline.
5. The study results indicate that particular attention should be paid to the problem of social and emotional functioning of RA patients and may be helpful in building therapeutic strategies and further research on HRQoL.
